# Association of folate intake and plasma folate level with the risk of breast cancer: a dose-response meta-analysis of observational studies

**DOI:** 10.18632/aging.103881

**Published:** 2020-11-04

**Authors:** Xueting Ren, Peng Xu, Dai Zhang, Kang Liu, Dingli Song, Yi Zheng, Si Yang, Na Li, Qian Hao, Ying Wu, Zhen Zhai, Huafeng Kang, Zhijun Dai

**Affiliations:** 1 Department of Breast Surgery, The First Affiliated Hospital, College of Medicine, Zhejiang University, Hangzhou, China; 2 Department of Oncology, The Second Affiliated Hospital of Xi’an Jiaotong University, Xi’an, China; 3 Department of Hepatobiliary Surgery, The First Affiliated Hospital of Xi'an Jiaotong University, Xi'an, China

**Keywords:** breast cancer, risk, folate, dose-response, meta-analysis

## Abstract

Epidemiological studies showing the correlation between folate and the breast cancer risk have revealed inconsistent results. Hence, we conducted a dose-response meta-analysis of observational studies to obtain more reliable conclusions. We searched PubMed and Embase for studies published before April 2019 and identified 39 studies on folate intake and 12 studies on plasma folate level. The combined odds ratios (ORs) and 95% confidence intervals (CIs) were extracted to estimate the breast cancer risk. Folate intake was inversely correlated with the breast cancer risk when the highest and lowest categories (OR = 0.85, 95% CI = 0.79–0.92) were compared, and the dose-response result showed that folate intake had a linear correlation with the breast cancer risk. Moreover, a higher folate intake correlated with a lower breast cancer risk in premenopausal women (OR = 0.80, 95% CI = 0.66–0.97), but not in postmenopausal women (OR = 0.94, 95% CI = 0.83–1.06). However, plasma folate levels were not correlated with the breast cancer risk (OR = 0.98, 95% CI = 0.82–1.17). Folate intake was negatively correlated with the breast cancer risk; however, its practical clinical significance requires further study. Furthermore, additional folate supplements should be considered carefully.

## INTRODUCTION

Breast cancer has a global annual incidence of >2 million cases [1]. The incidence of breast cancer is the highest in women and is the main cause of cancer-related deaths in women worldwide [1]. Research shows that from 1990 to 2017, the incidence and mortality of breast cancer continued to rise, and the burden of breast cancer continued to increase globally [2]. There are numerous causes for the observed increase in the incidence of malignancies, such as increase in the number of women participating in screening programs, poor diet, and inadequate physical activity [3]. The need for preventive measures such as promoting healthy eating habits is also worth highlighting [3–5].

Folate, also known as vitamin B9, is present in many foods. It is essential for the regeneration of methionine, which is needed for DNA methylation, a process that synthesizes purine and pyrimidine thymidine for DNA repair [6]. Studies [7–11] have suggested that in carcinogenic processes, folate participates in the so-called one-carbon metabolic pathway. This pathway is crucial for DNA synthesis, repair, and methylation [6]. In many cancers such as prostate cancer [12, 13] and breast cancer [14], epigenetic changes (such as DNA hypomethylation and hypermethylation), DNA uracil mismatch, and chromosome rearrangement have been observed. These findings indicate that changes in vitamin B9 levels might influence cancer progression.

According to epidemiological studies, folate intake is inversely correlated with the mortality risk associated with esophageal squamous cell carcinoma [15] and may be related to a reduction in the risk of colorectal cancer [16]. In the past few years, the correlation between folate levels and the risk of breast cancer has been a major concern. However, the findings of recently published meta-analyses are inconsistent [17, 18]. The meta-analysis by Zhang et al. has indicated little correlation between folate intake and the risk of breast cancer [19]. In another meta-analysis, Tio et al. have reported that the risk of breast cancer might not be related to folate intake and that this risk did not change with menopause or hormone receptor status [20]. Conversely, Chen et al. have suggested a negative correlation between folate intake and the risk of breast cancer [21]. Hence, to obtain more credible conclusions, we performed a dose-response analysis to measure the risk of breast cancer by incrementally increasing the folate level. To the best of our knowledge, no meta-analysis has evaluated the correlation between plasma folate level and the risk of breast cancer. Therefore, we evaluated the correlation of folate intake and plasma folate level with the risk of breast cancer in a dose-response meta-analysis on the basis of eligible observational research studies.

## RESULTS

### Study selection and features

**Figure 1:**
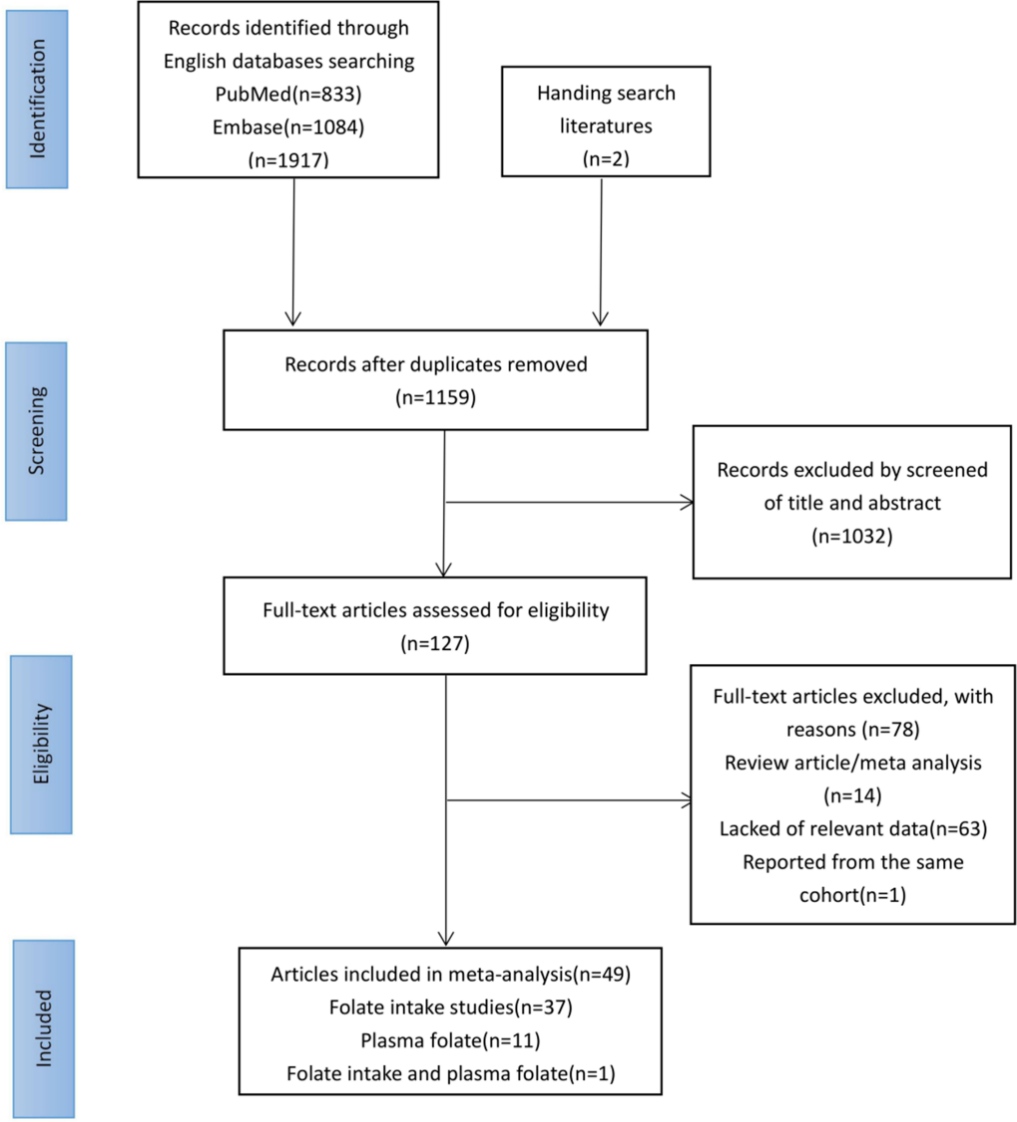
**Flowchart of included studies for the meta-analysis.**

Figure 1 shows a flowchart of our study selection process. First, we identified 1,919 articles using PubMed and Embase databases and manual searches. Second, we excluded 760 duplicate articles, 1,032 articles that lacked relevance by reading the title and abstract in detail, 14 review/meta-analysis articles, 63 articles that did not provide enough information (folate dosage or the number of cases/controls/persons or OR/HR/RR), and 1 article with the same cohort. Eventually, 49 articles describing 51 observational studies met the inclusion criteria, of which 2 articles contained 2 separate studies [22, 23]. One study by Gong examined the association between the risk of breast cancer and dietary folate among African Americans and European Americans in separate analyses. Another article published by Lin studied the relationship between the risk of breast cancer and both serum folate and dietary folic acid concentrations. The features of the included studies regarding folate intake are listed in Supplementary Table 2, and the features of the included studies regarding plasma folate level are listed in Supplementary Table 1.

Based on the inclusion and exclusion criteria, we included 39 related studies to determine the correlation of folate intake and the risk of breast cancer. Of the 39 studies, 19 were prospective cohort studies [9, 22–38], which included 37,917 cases, and 20 were case-control studies [22, 25–29, 39], which included 13,074 cases and 17,497 controls. Of the included studies, 12 [10, 24, 31, 33, 35, 38, 40–45] were conducted in Europe, 19 [9, 11, 22, 23, 26, 32, 36, 37, 39, 46–54] were conducted in Americas, 6 [27–30, 34, 55] in Asia, and 1 [56] was conducted in Australia. Of the 39 studies, 13 studies [22, 23, 27, 29, 32, 35, 38, 40, 41, 50–53] were stratified by menopausal status and provided risk estimates.

Based on the inclusion and exclusion criteria, we analyzed 12 relevant studies to examine the correlation between plasma folate level and the risk of breast cancer, including 10 case-control studies [39, 57–65], consisting of 7850 cases and 8898 controls, and 2 cohort studies [66, 67], consisting of 815 incident cases. Of them, 4 were conducted in Europe [59, 60, 62, 66], 5 were conducted in America [23, 57, 58, 65, 67], 1 were conducted in Asia [63], 1 were conducted in Uganda [61], and 1 was conducted in Australia [64]. Moreover, among the 12 studies, 7 [23, 57–60, 62, 67] were stratified by menopausal status and provided risk estimates. The risk estimates in most studies were adjusted for underlying confounding factors, including patient age, body mass index (BMI), educational level, parity, age at first birth, age at menarche, age at menopause, history of breast diseases, smoking, and alcohol intake. Supplementary Tables 1 and 2 present the adjusted confounding factors.

### Folate intake and the risk of breast cancer

**Figure 2:**
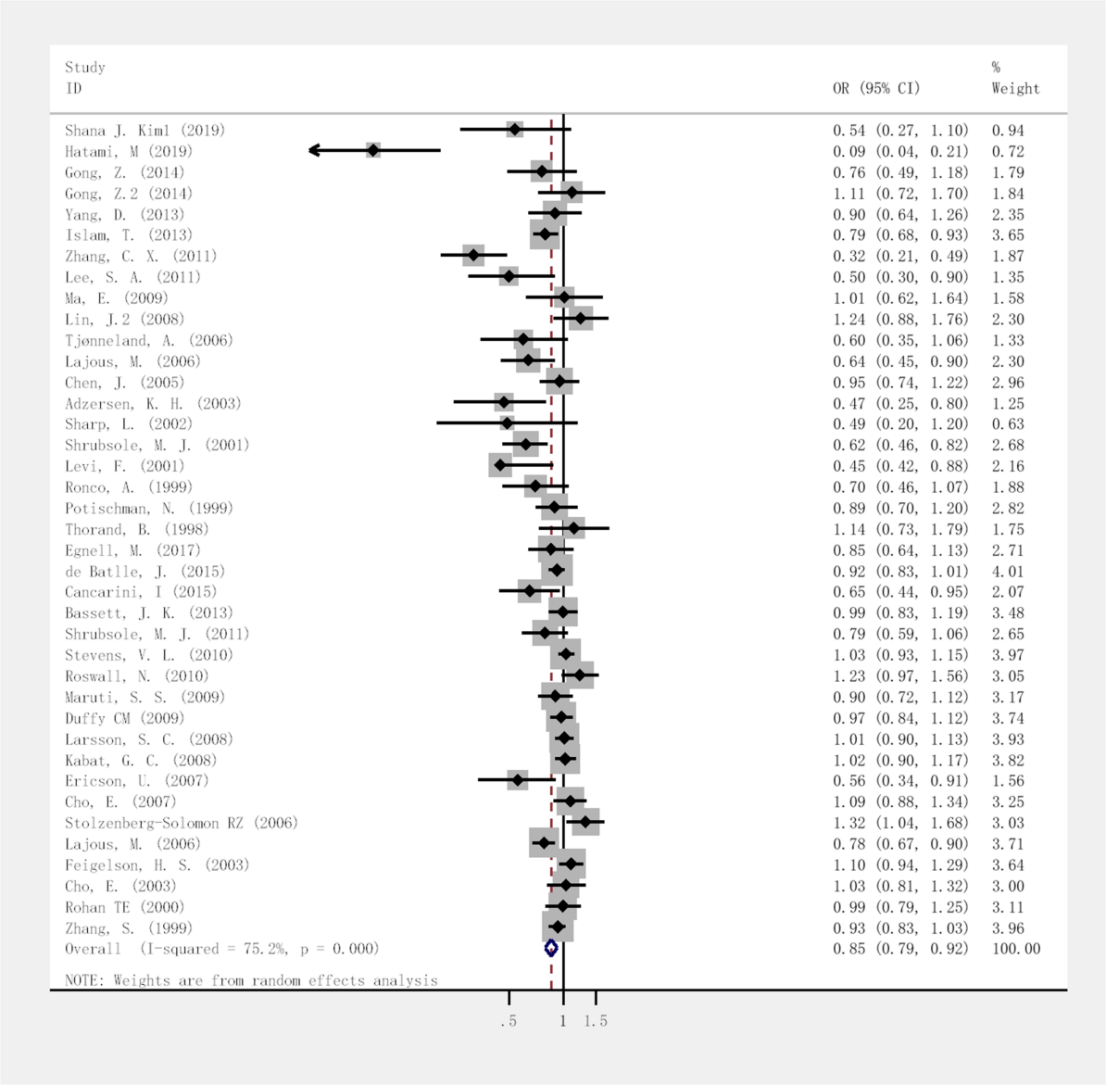
**Forest plot of meta-analysis of breast cancer risk in relation to highest vs lowest categories of folate intake.** Note: Weights are from random-effects analysis. Abbreviations: OR, odds ratio; CI, confidence interval.

**Figure 3:**
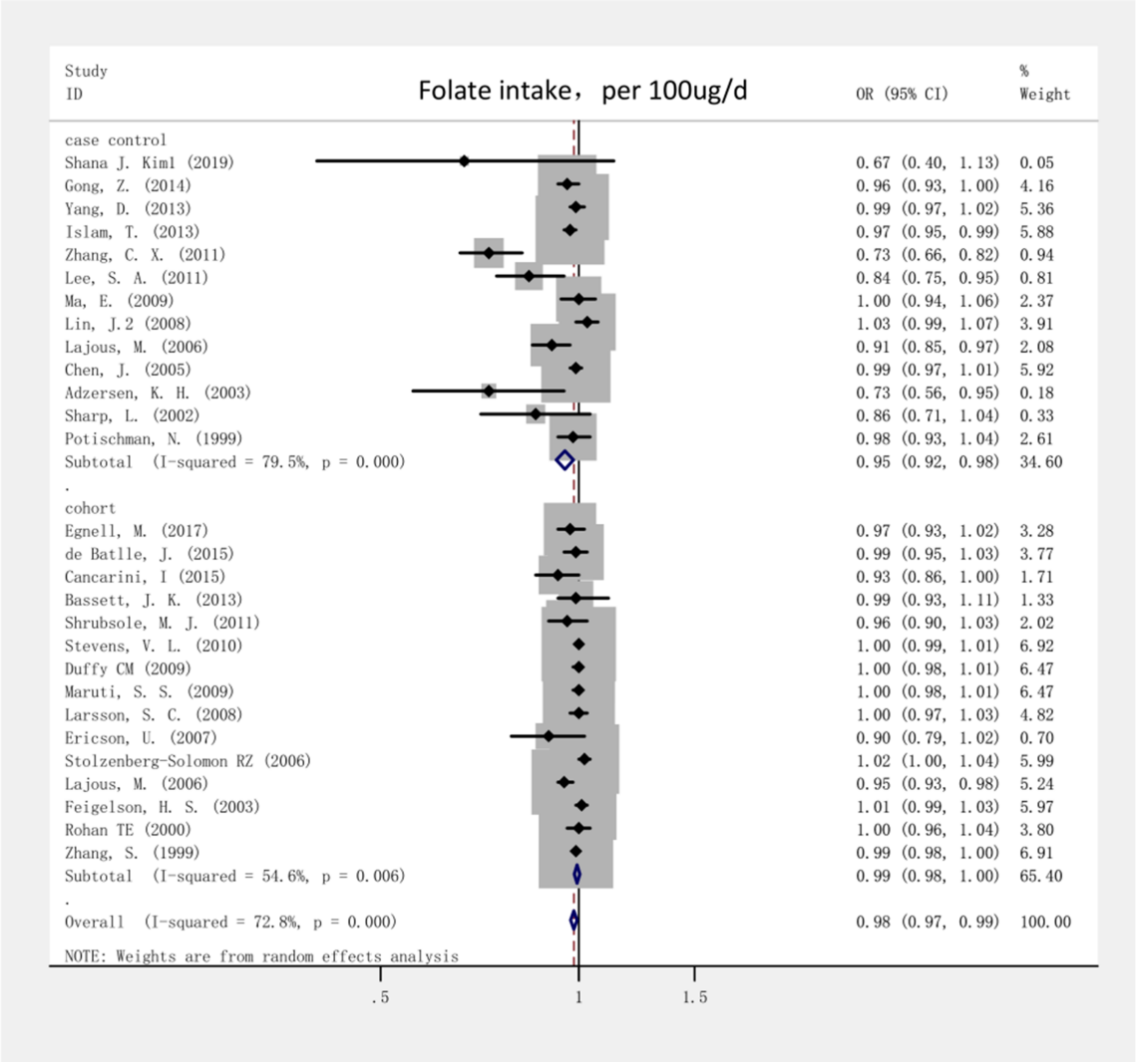
**Forest plot of meta-analysis of the association between folate intake increment (per 100ug/day) and breast cancer risk.** Note: Weights are from random-effects analysis. Abbreviations: OR, odds ratio; CI, confidence interval.

**Figure 4:**
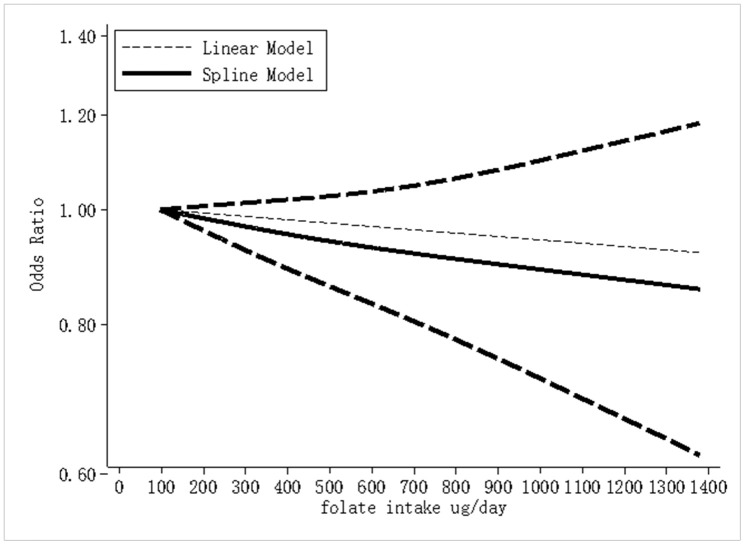
**Dose-response meta-analysis of folate intake and breast cancer risk (linear and nonlinear models).**

The highest and lowest folate intake levels negatively correlated with the risk of breast cancer, with a combined OR of 0.85 (95% CI, 0.79–0.92; *I*^2^ = 75.2%, P < 0.001; Figure 2). For the case-control and cohort studies, the combined ORs were 0.68 (95% CI, 0.57–0.81; *I*^2^ = 76.3%; P < 0.001) and 0.97 (95% CI, 0.91–1.03; *I*^2^ = 53.3%; P = 0.316), respectively. We analyzed 13 case-control studies and 15 cohort studies that met the selection criteria to determine the dose-response correlation of folate intake with the risk of breast cancer. For every 100-μg/day increase in folate intake, the combined OR for the risk of breast cancer was 0.98 (95% CI, 0.97–0.99; *I*^2^ = 72.8%; P = 0.002; Figure 3). The summary OR was 0.95 (95% CI, 0.92–0.98) for the case-control studies and 0.99 (95% CI, 0.98–1.00) for the cohort studies. Fifteen eligible cohort studies showed a linear dose-response correlation between folate intake and the risk of breast cancer (P = 0.0667; Figure 4), indicating that for every 100- μg/day increase in folate intake, the risk of breast cancer was reduced by 2%. The funnel plots (Supplementary Figure 1) as well as the Begg (P = 0.003) and Egger test results (P = 0.001) indicated obvious publication bias among the considered studies. Moreover, as shown in the sensitivity analysis, the OR ranged from 0.97 to 0.99 and a single study had no influence on the results, indicating that our outcomes were statistically robust (Supplementary Figure 2).

The subgroup analysis was stratified by study types, menopausal status, geographic location, receptor tumor status, and follow-up time. The outcomes were presented in Table 1. Stratification by menopausal status showed that a higher folate intake might correlate with a lower risk of breast cancer in premenopausal women (OR = 0.80; 95% CI, 0.66–0.97; P = 0.022), but not in postmenopausal women (OR = 0.94; 95% CI, 0.83–1.06; P = 0.320). In the *ER+* (OR = 0.78; 95% CI, 0.65–0.94) and *ER−* breast cancer subtypes (OR = 0.71; 95% CI, 0.56–0.90), folate intake was negatively correlated with the incidence of breast cancer, but not in the *PR+, PR−, ER+/PR+, ER−/PR−, HER2+, and HER2−* subtypes. In subgroup analyses by geographic location, the result showed a negative correlation between folate intake and the risk of breast cancer in Asian (OR = 0.65; 95% CI, 0.49–0.84) and European women (OR = 0.79; 95% CI, 0.68–0.92).

**Table 1:** Subgroup analyses of folate intake and breast cancer.

**Analysis specification**	**No. of studies**	**OR(95% CI)**	**Heterogeneity**		**P**
			**I 2**	**P**	
Highest vs lowest					
All studies	39	0.85(0.79-0.92)	75.2%	0.000	0.000
Case-control	20	0.68(0.57-0.81)	76.3%	0.000	0.000
Cohort	19	0.97(0.91-1.03)	53.3%	0.003	0.316
Increment of 100 ug/d					
All studies	28	0.98(0.97-0.99)	72.8%	0.000	0.002
Case-control	13	0.95(0.92-0.98)	79.5%	0.000	0.001
Cohort	15	0.99(0.98-1.00)	54.6%	0.006	0.025
Menopausal status					
Premenopausal	10	0.80(0.66-0.97)	59.7%	0.006	0.022
Postmenopausal	14	0.94(0.83-1.06)	62.2%	0.001	0.320
Receptor tumor status					
ER+	10	0.78(0.65-0.94)	69.0%	0.001	0.009
ER-	10	0.71(0.56-0.90)	42.6%	0.074	0.005
PR+	4	0.67(0.41-1.10)	88.5%	0.000	0.113
PR-	4	0.83(0.68-1.02)	0.0%	0.399	0.083
ER+/PR+	9	0.92(0.80-1.07)	72.1%	0.000	0.284
ER-/PR-	9	0.99(0.94-1.05)	0.0%	0.679	0.837
HER2+	3	0.86(0.58-1.28)	0.0%	0.891	0.446
HER2-	3	0.87(0.64-1.18)	47.2%	0.150	0.360
Source folate					
Dietary	35	0.86(0.79-0.93)	70.4%	0.000	0.000
Supplement	7	1.05(0.95-1.17)	20.6%	0.273	0.326
Dietary+Supplement	11	0.99(0.89-1.10)	55.9%	0.012	0.882
Geographic location					
Europe	12	0.79(0.68-0.92)	75.0%	0.000	0.002
America	19	0.99(0.93-1.06)	42.4%	0.027	0.817
Asia	6	0.65(0.49-0.84)	75.4%	0.001	0.001
Follow-up duration					
<10 years	9	0.93(0.82-1.06)	67.5%	0.002	0.275
≥10 years	10	1.00(0.94-1.07)	49.2%	0.039	0.937

Abbreviations: OR, odds ratio; CI, confidence interval.

### Plasma folate level and the risk of breast cancer

**Figure 5:**
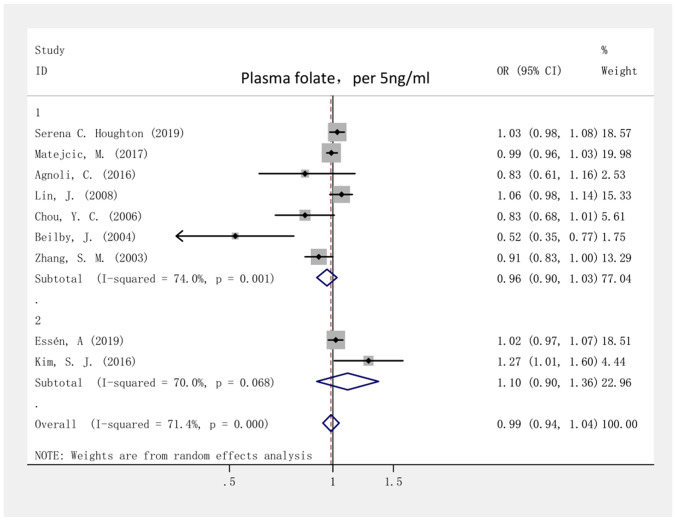
**Forest plot of meta-analysis of the association between plasma folate increment (per 5ng/ml) and breast cancer risk.** Note: Weights are from fixed-effects analysis. Abbreviations: OR, odds ratio; CI, confidence interval.

The highest and lowest plasma folate levels had no correlation with the risk of breast cancer, with a combined OR of 0.98 (95% CI, 0.82–1.17; *I*^2^ = 63.0%; P = 0.822; (Supplementary Figure 3). For the case-control and cohort studies, the summary ORs were 0.93 (95% CI, 0.77–1.13; *I*^2^ = 63.4%; P = 0.488) and 1.63 (95% CI, 0.61–4.37; *I*^2^ = 67.9%; P = 0.331), respectively. On the basis of the selection criteria, 7 case-control research studies and 2 cohort research studies were chosen for the dose-response analysis of the correlation between plasma folate level and the risk of breast cancer. Figure 5 shows that for every 5-ng/ml increase in the plasma folate level, the summary OR for the risk of breast cancer was 0.99 (95% CI = 0.94–1.04; *I*^2^ = 71.4%; P = 0.654), indicating that a 5-ng/ml increase in the plasma folate level had no relationship the with the risk of breast cancer. Funnel plots (Supplementary Figure 4) as well as the Begg (P = 0.466) and Egger test (P = 0.269) results indicated that there was no significant publication bias. The ORs ranged from 0.94 to 1.04 in the sensitivity analyses (Supplementary Figure 5), indicating that our results were statistically stable.

Table 2 presents the results of subgroup analysis. When the subgroup analysis was stratified by study types, menopausal status, geographic location, receptor tumor status, and follow-up time, the results were stable.

**Table 2:** Subgroup analyses of plasma folate and breast cancer.

**Analysis specification**	**No. of studies**	**OR(95% CI)**	**Heterogeneity**		**P**
			**I 2**	**P**	
Highest vs lowest					
All studies	12	0.98(0.82-1.17)	63.0%	0.002	0.822
Case-control	10	0.93(0.77-1.13)	63.4%	0.003	0.488
Cohort	2	1.63(0.61-4.37)	67.9%	0.078	0.331
Increment of 5 ng/ml					
All studies	9	0.99(0.94-1.04)	71.4%	0.000	0.654
Case-control	7	0.96(0.90-1.03)	74.0%	0.001	0.246
Cohort	2	1.10(0.90-1.36)	70.0%	0.068	0.346
Menopausal status					
Premenopausal	6	1.05(0.89-1.24)	24.1%	0.253	0.593
Postmenopausal	6	0.98(0.86-1.11)	17.5%	0.300	0.722
Receptor tumor status					
ER+	3	1.11(0.72-1.71)	78.6%	0.009	0.649
ER-	3	0.94(0.68-1.30)	0.0%	0.455	0.698
PR+	3	1.07(0.67-1.71)	77.6%	0.012	0.776
PR-	3	1.02(0.79-1.32)	0.0%	0.987	0.864
ER+/PR+	2	1.26(0.80-1.98)	72.3%	0.057	0.314
ER-/PR-	2	1.08(0.69-1.68)	0.0%	0.642	0.750
HER2+	2	1.18(0.74-1.89)	0.0%	0.885	0.486
HER2-	2	1.02(0.74-1.40)	23.1%	0.254	0.912
Geographic location					
Europe	4	1.00(0.86-1.17)	14.4%	0.320	0.976
America	5	1.10(0.83-1.46)	64.8%	0.023	0.505
Asia	1	0.52(0.26-1.04)	.%	.	0.066

Abbreviations: OR, odds ratio; CI, confidence interval.

## DISCUSSION

In recent years, researchers have increasingly examined the effect of folate level on the risk of breast cancer, but inconsistent results have been reported. Two meta-analyses have reported that folate intake does not significantly decrease the risk of breast cancer [19, 20].

However, other studies have shown different results [21, 68]. In a meta-analysis, folate intake was negatively correlated with the risk of breast cancer [21], while in another meta-analysis, folate intake did not decrease the total incidence of breast cancer, but it reduced the risk for the *ER−* subtype, especially the *ER−/PR−* subtypes [68]. In contrast, in a dose-response meta-analysis conducted in 2014, folate intake had a J-type dose-response correlation with the risk of breast cancer [19]. Therefore, on the basis of the inconsistencies observed in previous meta-analyses and systematic reviews, we included new cohort studies [10, 40, 45, 46, 56] to strengthen our investigation on the correlation between folate intake and the risk of breast cancer. Thus far, no meta-analysis or dose-response research has investigated the correlation of plasma folate level and the risk of breast cancer. Hence, we conducted this study to establish a more definitive correlation between the risk of breast cancer and both folate intake and plasma folate level.

In our study, 39 observational studies on folate intake and the risk of breast cancer were included for meta-analysis. Our study revealed that folate intake had an inverse correlation with the risk of breast cancer. Moreover, the results of the dose-response analysis suggest that this correlation was linear. We also found that every 100-μg/day increase in folate intake could influence the reduction of the risk of breast cancer by 2%.

The results of the subgroup analysis also showed that a higher folate intake might be correlated with a lower incidence of breast cancer in premenopausal women, but not in postmenopausal women. The discrepancy between folate insufficiency and sufficiency might be a reason for the existing correlation between folate intake and the incidence of breast cancer in premenopausal women [55]. Premenopausal women, owing to their fertility, may have a greater demand for folate than postmenopausal women. As shown in the subgroup analysis, folate intake was negatively correlated with the incidences of *ER+* and *ER−* breast cancer subtypes. However, in the *PR+, PR−, ER+/PR+, ER−/PR−, HER2+,* and *HER2−* subtypes, this relationship was not statistically significant because of the relatively small number of cases. Low folate levels lead to methyl deficiency, which could be related to the methylated ER gene *CpG island*. ER *CpG island* methylation correlates with ER gene expression deficiency in *ER−* breast cancer [69–73]. Our results are consistent with the hypothesis that folate intake negatively correlates with *ER−* breast cancer. Nevertheless, we also found a similar correlation between folate intake and *ER+* breast tumors. This outcome was contrary to our expectation or may have been accidental; hence, further studies with larger samples are needed to clarify this outcome in future research.

Triple-negative breast cancer (TNBC; *ER−/PR−/HER2−*) accounts for 10%–20% of all cases of breast cancer and is an invasive disease having no valid targeted therapeutic method [74, 75]. However, to the best of our knowledge, only few studies have studied the TNBC subgroup, and the correlation of folate level with TNBC incidence is worthy of intensive future research.

Furthermore, when our analysis was stratified by geographic location, the results showed that in Europe and Asia, folate intake had an inverse correlation with the incidence of breast cancer. This may be because compared to the recommended daily intake of 400 μg dietary folate equivalent (DFE) for adults [76], the folate intake in the present American population may be sufficient to saturate the metabolic system. The average daily folate intake of women with and without supplementary folate intake in America was 665 μg DFE and 1013 μg DFE, respectively [77]. These variations may be related to the dietary composition and genetic susceptibility in different regions. Moreover, we found that increased folate intake from dietary sources was linked to a reduced risk of breast cancer, but the same was not true for folate from supplements. Therefore, we infer that diets may contain complicated components of a series of bioavailable ingredients and that folate interacts with these ingredients to reduce the risk of breast cancer. However, it is difficult to determine whether the anticancer effect is due to folate intake or the interaction between folate and other nutrients. Further research is needed to determine whether folate intake has clinical significance in reducing the risk of breast cancer, and the use of additional folate supplements should be carefully considered.

In previous original studies, 10 case-control studies [23, 57, 59–67, 78] and 2 prospective cohort studies [66, 67] examined the effect of plasma folate on the incidence of breast cancer, with inconsistent results. Several studies have also revealed that plasma folate level may be positively correlated with the risk of breast cancer. A cohort study based on data from the Swedish Apolipoprotein-related Mortality Risk (AMORIS) cohort in 2019 has shown that high fasting plasma folate levels might increase the risk of breast cancer [66]. Another study has revealed that elevated plasma folate levels might increase the risk of *BRCA1/2* mutation [39]. Conversely, a case-control study by Beilby [64], which included 141 cases and 109 controls, has shown that elevated plasma folate levels might have a correlation with decreased the incidence of breast cancer in the total population. In another prospective nested case-control study [65], plasma folate level had an inverse correlation with the incidence of breast cancer.

In our meta-analysis, 12 observational studies on plasma folate level and the risk of breast cancer were included. To the best of our knowledge, our study was the first to conduct a meta-analysis on the correlation between plasma folate level and the risk of breast cancer, but we were unable to establish any apparent correlation. Further dose-response analysis also did not establish a correlation between 5 ng/ml increments in plasma folate levels and the risk of breast cancer. Nevertheless, our results indicated statistical stability when stratified by study types, menopausal status, geographic location, receptor tumor status, and follow-up time.

Despite some related hypotheses, no precise mechanism has been found to clarify the link between folate level and the incidence of breast cancer. We believe that the role of folate in the so-called one-carbon metabolism pathway has a potential action in carcinogenesis. First, folate, 5-methyltetrahydrofolate, transforms homocysteine to methionine and then to S-adenosylmethionine [79]. The latter is a ubiquitous methyl donor, usually providing methyl for methylation reactions, especially in DNA and RNA biosynthesis [79–82]. Folate, as a nutrient for one-carbon metabolism, affects DNA methylation by regulating the S-adenosylmethionine level. Deletion of S-adenosylmethionine could cause DAN hypomethylation, which induces the expressions of proto-oncogenes and ultimately leads to cancer [83]. Folate insufficiency also causes the methylation of uracil into thymine, resulting in the incorporation of uracil into DNA [80]. This incorporation further causes chromosome breakage and carcinogenesis [79, 83]. However, future studies must be conducted to verify these hypotheses through in-depth research on carcinogenesis mechanisms.

Our meta-analysis had significant heterogeneity. To find the source of this heterogeneity, we conducted subgroup analyses based on menopausal status, receptor tumor status, geographic location, and folate intake sources. However, no significant source of heterogeneity was identified. Therefore, we calculated the summary OR with the 95% CI using the random-effects model to reduce deviations in the association. In the sensitivity analysis, any separate study did not affect the combined OR, which showed that our results were robust.

This study had limitations. First, many included studies were case-control studies; hence, recall and selection biases inevitably influenced the study results. Second, an evident heterogeneity was found in our research. Despite performing a subgroup analysis, we failed to identify the source of this heterogeneity. We speculate that the heterogeneity might have been caused by other factors such as the interaction between nutrients and population baseline characteristics. Third, since breast cancer is a heterogeneous disease, only specific subtypes might be affected by folate level. Finally, our research had a significant publication bias, which may have been caused by the relative ease of publishing studies with positive results, compared to those with negative results.

Nevertheless, this study also has its strengths. First, compared with previous studies, our meta-analysis included more original studies with larger sample sizes. Therefore, the conclusion from this meta-analysis could be considered more representative of actual conditions. Second, to the best our knowledge, the correlation between plasma folate level and the risk of breast cancer was examined for the first time in our study. Finally, this study covered a wide range of folate intake levels; hence, it could more accurately assess the dose-response correlation of folate intake with the risk of breast cancer. However, more elaborate studies with wider ranges of doses and time points are still necessary to further clarify this association.

In conclusion, our study results appear support the negative correlation between folate intake and the risk of breast cancer. A dose-response meta-analysis revealed that every 100 μg/day increase in folate intake contributed to a 2% reduction in the risk of breast cancer. However, our study showed that plasma folate intake itself had no correlation with the risk of breast cancer. Therefore, whether folate intake has practical clinical significance requires further study, and the use of additional folate supplements should be carefully considered.

## MATERIALS AND METHODS

### Search strategy

Articles published before April 2019 estimating the correlation of folate intake and the risk of breast cancer were retrieved from two electronic databases (PubMed and Embase). Articles were retrieved using the following keywords: (“folic acid” OR “vitamin M” OR “vitamin B9” OR “folate” OR “folvite”) AND (“breast neoplasm” OR “breast tumor” OR “breast cancer” OR “mammary cancer” OR “breast carcinoma”). To screen for other qualified studies, all relevant original articles as well as the bibliographies of review articles were manually searched. Three authors independently read the retrieved literature, screened the relevant publications according to the exclusion criteria, and then removed any duplicated articles. Disagreement among three authors was resolved by discussion.

### Selection criteria

Studies that met the following inclusion criteria were included in the analysis: 1) an original observational study (cohort or case-control); 2) used folate as the exposure factor and breast cancer as outcome; 3) provided risk estimation as OR, hazard ratio (HR), or relative risk (RR), and the corresponding 95% confidence interval (CI) or sufficient data for estimation (all results were represented by OR); and 4) provided the number of cases and controls or person-years for every folate dose group (or data available for calculation) for a dose-response analysis. Only the latest and content-rich studies were included if the research pertained to the same or overlapping cohorts. Studies were excluded if they were meta-analyses and if they provided insufficient data.

### Data extraction and quality assessment

Each full report was reviewed to confirm its qualification based on the inclusion criteria, and all relevant data (year of publication, first author, study type, age, geographic location, years of follow-up, cases/controls/person-years, folate exposure assessment, comparative categories, menopausal status, receptor tumor status, and wholly adjusted model covariates) were independently extracted and tabulated in Supplementary Tables 2 and 1. When more than one multivariable-adjusted effect estimate was observed in the studies, we chose the maximum adjusted effect estimate for potential confounding factors. If the results of both dietary and total intakes (dietary intake plus supplementation) were provided, the total folate intake was extracted. The quality of each selected publication was assessed independently by three authors according to the Newcastle-Ottawa Quality Assessment Scale (NOS) [84]. The content of the studies was evaluated for four major aspects: selection, comparability, exposure, and results, and thereafter, were categorized into high, medium, and low quality. A study with a score >6 was considered a good-quality study.

### Statistical analyses

The *Q* and *I*^2^ statistics were combined to quantify data heterogeneity. The *I*^2^ statistics were used to explain the research variability caused by the heterogeneity, rather than by chance. When the *Q* statistic P value was <0.05 or I^2^ was >50%, heterogeneity was evident in the studies. When the heterogeneity was significant, the random-effects model was used; otherwise, the fixed-effects model was used. If a combination of clinical issues was apparent, the fixed-effects model could be accepted [85]. To identify the sources of heterogeneity, subgroup analyses were performed by analyzing menopause status, receptor tumor status, geographic location, length of follow-up, and folate sources.

We assessed the relationship between folate intake and the risk of breast cancer by combining OR values and 95% CIs. First, the random-effects model was used to count the highest and lowest categories of the combined ORs and 95% CIs for folate intake. Second, for trend estimation, the generalized least square was used and the risk estimates for specific categories were converted to OR estimates for a 100 μg/day increase in folate intake. We assumed that the correlation between the natural logarithm of OR and the increase in folate intake was linear and then calculated the estimates [86]. The midpoint of the closed interval was assigned as the value for each folate category. For the upper open interval, we multiplied the value of the interval endpoint by 1.5 and the value of the lower open interval by 0.5 (folate intake was assumed to be normally distributed) [19]. The outcome of the random-effects meta-analysis was applied to combine the ORs for increased 100 μg/day during folate intake [87]. Third, a dose-response random-effects meta-analysis was performed for related natural logs of the ORs in all folate intake categories [86, 88]. The limited cubic splines with 3 knots were used to model folate and then obtain the dose-response curve.

To assess the underlying publication bias, visually inspected funnel plots for the risk of breast cancer was constructed. Moreover, the Egger [89] and Begg [90] tests were used to evaluate the effect of this publication bias on the risk of breast cancer. All P values were two-sided. The selected studies were considered statistically significant when the P value was <0.05. Stata Version 15.0 software (StataCorp, College Station, TX, USA) was used for the statistical analyses.

## Supplementary Material

Supplementary Figures

Supplementary Table 1

Supplementary Table 2
