# Supplementary material for: Association of folate intake and plasma folate level with the risk of breast cancer: a dose-response meta-analysis of observational studies
Source: Aging (Albany NY). 2020 Nov 4;12(21):21355–75. doi: 10.18632/aging.103881 (PMC7695428; doi:10.18632/aging.103881)
Supplement: Supplementary Figures [file aging-12-103881-s001..pdf]

SUPPLEMENTARY FIGURES

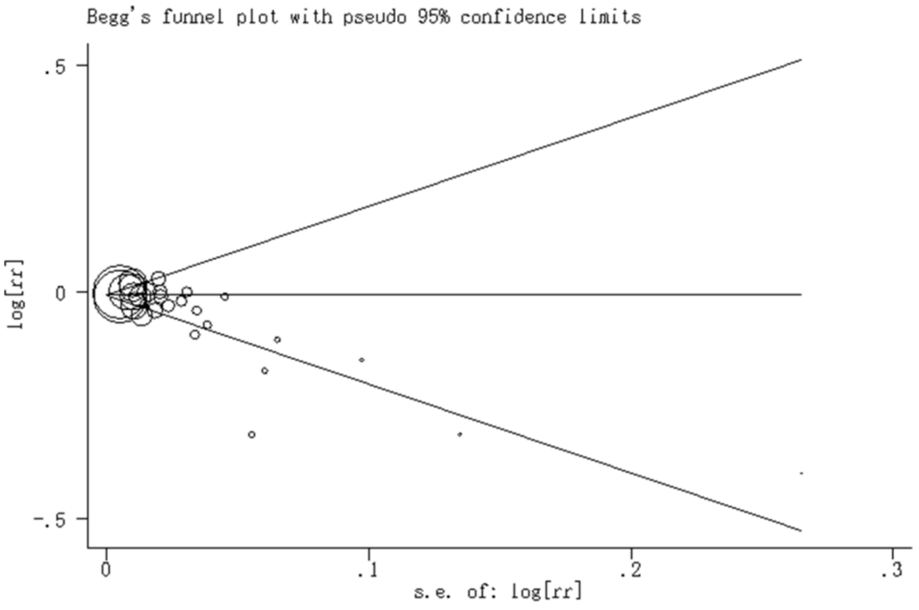

Supplementary Figure 1. Funnel plot of meta-analysis of breast cancer risk in relation to folate intake increment (per 100ug/day).

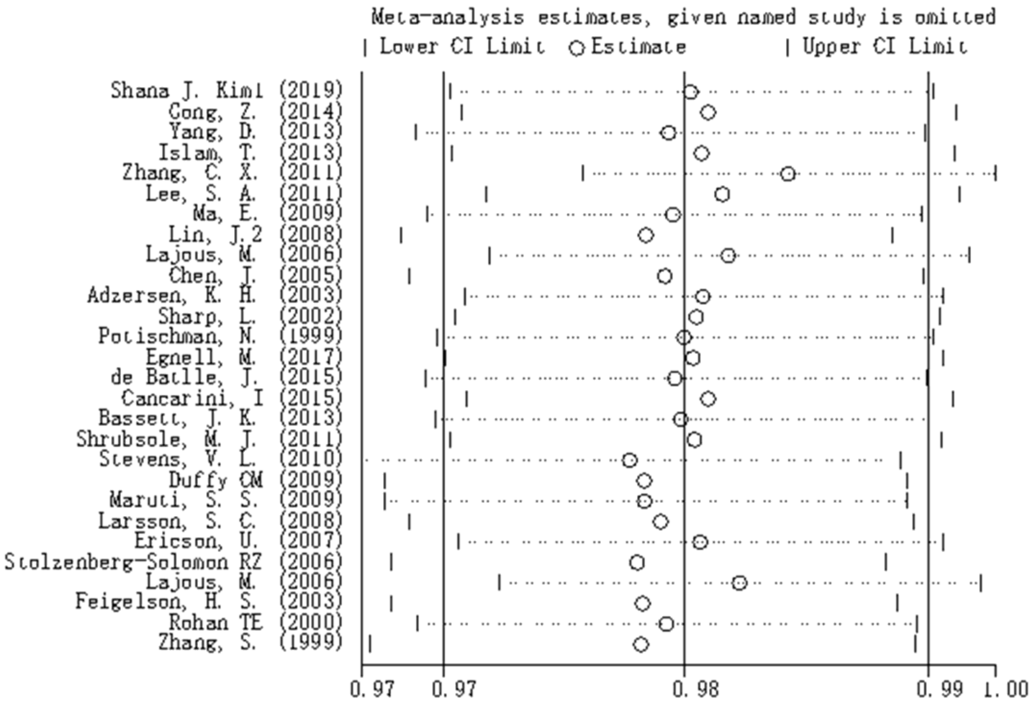

Supplementary Figure 2. Sensitivity analysis of meta-analysis of breast cancer risk in relation to folate intake increment (per 100ug/day).

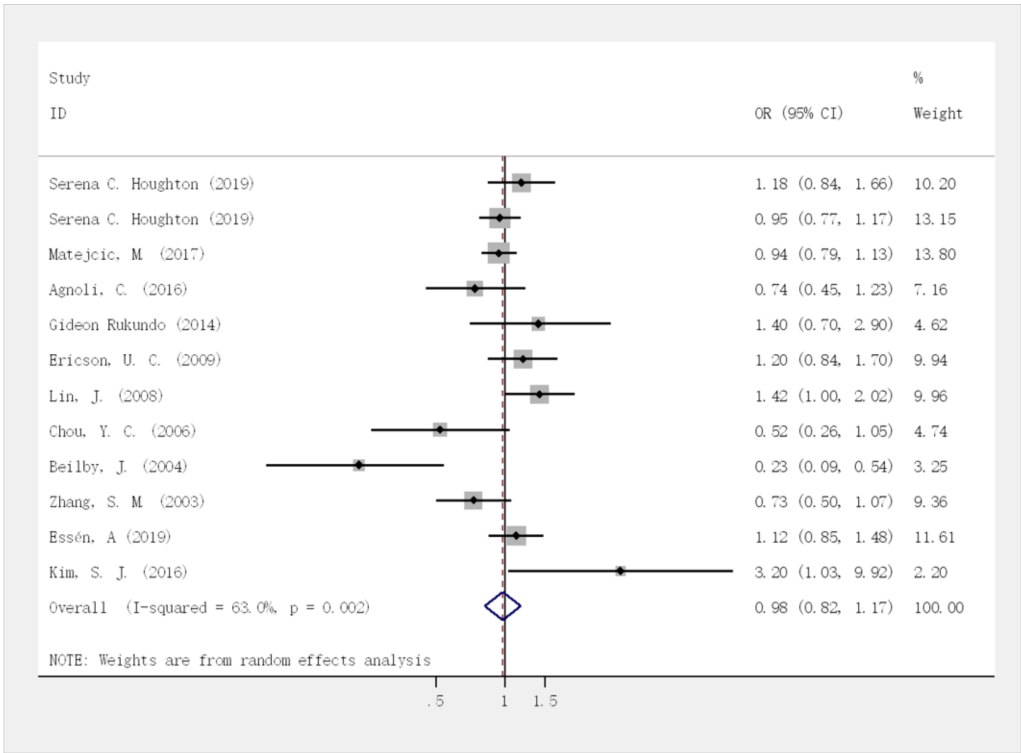

Supplementary Figure 3. Forest plot of meta-analysis of breast cancer risk in relation to highest vs lowest categories of plasma folate. Note: Weights are from random-effects analysis. Abbreviations: OR, odds ratio; CI, confidence interval.

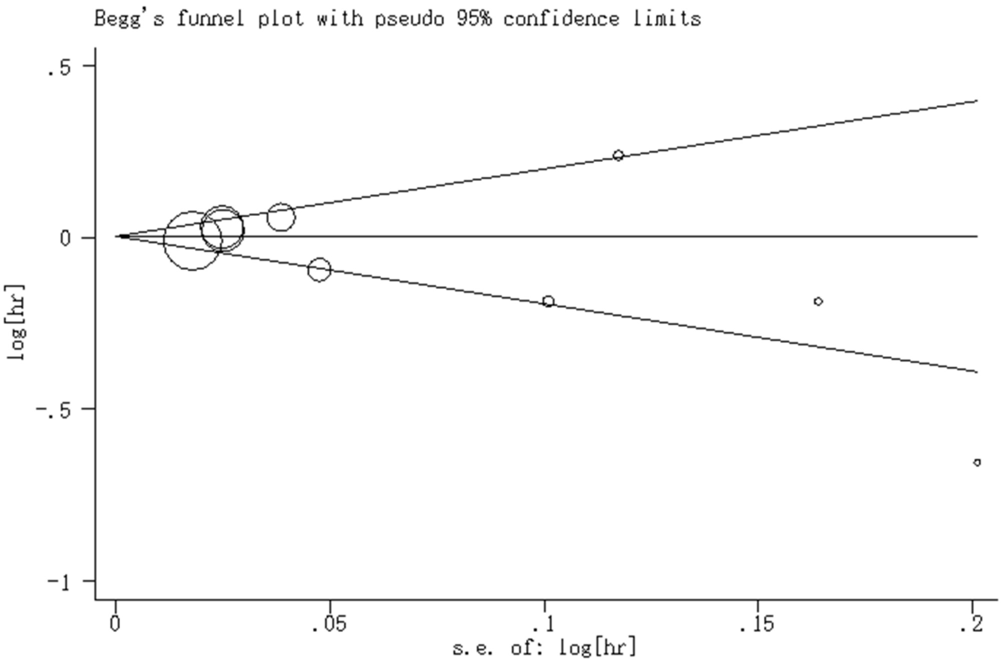

Supplementary Figure 4. Funnel plot of meta-analysis of breast cancer risk in relation to plasma folate increment (per 5ng/ml).

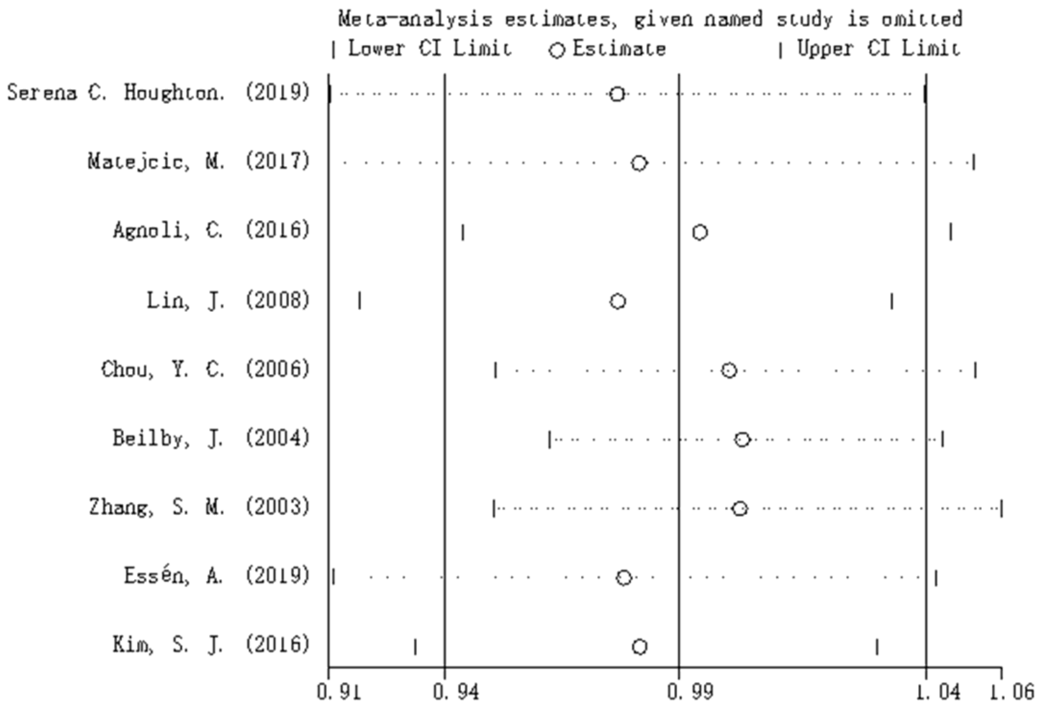

Supplementary Figure 5. Sensitivity analysis of meta-analysis of breast cancer risk in relation to plasma folate increment(per 5ng/ml).
