# Supplementary material for: Association of folate intake and plasma folate level with the risk of breast cancer: a dose-response meta-analysis of observational studies
Source: Aging (Albany NY). 2020 Nov 4;12(21):21355–75. doi: 10.18632/aging.103881 (PMC7695428; doi:10.18632/aging.103881)
Supplement: Supplementary Table 1 [file aging-12-103881-s002..pdf]

SUPPLEMENTARY TABLE

Supplementary Table 1. Features of studies included in the meta-analysis of plasma folate and breast cancer risk.

author	country	study type	Follow-up period (year)	Age(year)	Number of cases/controls/persons	plasma folate (ng/mL)	Adjusted OR(95% CI)	Adjustment factors
Serena C. Houghton.2019	US	Case–control	4.75	32-53	610/1207	≥23.7 VS <10.2	1.18(0.84-1.66)	matching factors, age at menarche, parity/age at first birth, history of breast cancer in mother or a sister, history of benign breast disease, height, body mass index at age 18, weight change since 18, and alcohol intake.
Serena C. Houghton.2019	US	Case–control	1990-2006	30-55	2241/2241	≥15.6 VS <4.7	0.95(0.77-1.17)	age at menarche, parity/age at first birth, age at menopause, family history of breast cancer in mother or a sister, history of benign breast disease, height, body mass index at 18, weight change from age 18, and alcohol intake at blood collection in 1990.
Matejcic, M.2017	Europe	Case–control	1992-2010	35-70	2491/2521	>19.80 VS <9.82	0.94(0.79-1.13)	date at blood collection, education, BMI, height, physical activity, ever use of hormone replacement therapy, alcohol intake, parity and age at first full-term birth combined, total energy intake and family history of breast cancer.
Agnoli, C.2016	Italy	Case–control	14.9	35-65	276/276	8.600-15.35 VS 2.609-5.968	0.74(0.45-1.23)	age, menopausal status, recruitment date, and distance between ORDET and EPIC recruitment, family history of breast cancer, age at menarche, parity, oral contraceptive use, smoking status, education, alcohol consumption, and BMI.
Gideon Rukundo.2014	Uganda	Case–control	2012.1-2012.5	>18	72/73	normal VS low	1.4(0.7-2.9)	age, parity, age at menarche and menopause, BMI, residence, use of hormonal contraception, tobacco use versus no tobacco usage, alcohol consumption, hemoglobin level.
Ericson, U. C.2009	Sweden	Case–control	1996-2004	55-73	313/626	17 VS 6	1.20(0.84-1.70)	age, blood-sampling date, weight, height, menopausal hormone therapy, age at menopause category, parity, household work category, socioeconomic status, smoking, total energy intake, and alcohol intake category.
Lin, J.2008	US	Case–control	1993-2004	≥45	848/848	>15.8 VS ≤5.1	1.42(1.00-2.02)	matching variables, age, randomized treatment assignment, BMI, family history of breast cancer in a

								first-degree relative, history of benign breast disease, smoking, physical activity, alcohol consumption, age at menarche, age at menopause, parity, and age at first birth.
Chou, Y. C.2006	Taiwan	Case–control	2004.1-2004.12	20-80	146/285	>14.42 VS <8.16	0.52(0.26-1.05)	sociodemographic characteristics, menstrual and reproductive history, menopausal status, lifestyle and medical history as well as family history of breast and other cancers.
Beilby, J.2004	Australia	Case–control	1992-1994	30-84	141/109	>9.0 VS <5.0	0.23(0.09-0.54)	C677T genotype, age of menarche, parity, alcohol intake, total fat intake.
Zhang, S. M.2003	America	Case–control	1989-1996	43-69	712/712	>14 VS <4.6	0.73(0.50-1.07)	age at menarche, parity, age at first birth, age at menopause, history of breast cancer in mother or a sister, history of benign breast disease, alcohol intake, body mass index at age 18y, body mass index at blood collection, and duration of postmenopausal hormone use.
Essén, A.2019	Sweden	Cohort	14	>20	795/19775	>32 VS <5	1.12(0.85-1.48)	age, education level, SES, CCI, serum glucose, triglycerides, cholesterol and fasting status.
Kim, S. J.2016	Canada	Cohort	6.3	18-70	20/164	>24.4 VS ≤24.4	3.20(1.03-9.92)	age at baseline and date of blood sample collection, BRCA mutation type, parity, baseline alcohol consumption, baseline regular smoker, and baseline regular coffee consumer.

Abbreviations: OR, odds ratio; CI, confidence interval; BMI,body mass index; BRCA, breast cancer; EPIC: european prospective investigation into cancer and nutrition; SES: socioeconomic status; CCI: platelet count increase index.
