# Supplementary material for: Association of folate intake and plasma folate level with the risk of breast cancer: a dose-response meta-analysis of observational studies
Source: Aging (Albany NY). 2020 Nov 4;12(21):21355–75. doi: 10.18632/aging.103881 (PMC7695428; doi:10.18632/aging.103881)
Supplement: Supplementary Table 2 [file aging-12-103881-s003..docx]

**Supplementary Table 2. Features of studies included in the meta-analysis of folate intake and breast cancer risk.**

| **Author** | **Country** | **Study type** | **Follow-up**  **period (year)** | **Age(year)** | **Number of cases/controls/persons** | **Folate intake(ug/day)** | **Adjusted**  **OR(95%CI)** | **Adjustment factors** |
| --- | --- | --- | --- | --- | --- | --- | --- | --- |
| Egnell, M.2017 | French | Cohort | 4.2 | ≥45 | 462/27,853 | >405.2 VS <265.8 | 0.85(0.64-1.13) | age, BMI, height, physical activity, smoking status, number of dietary records, alcohol intake, energy intake without alcohol, family history of cancer , educational level, number of biological children, menopausal status at baseline, and baseline use of hormonal treatment for menopause. |
| De Batlle, J.2015 | Europe | Cohort | 11.5 | 35-70 | 11575/367993 | >371 VS ≤221 | 0.92(0.83-1.01) | age at menarche, age at first full-term birth, menopausal status, weight and height, waist-to-hip ratio, smoking statu, educational attainment, physical activity, ever use of contraceptive pill and ever use of replacement hormones, alcohol intake, total dietary fiber, glycemic load, and ever use of vitamin or mineral supplements. |
| Cancarini, I.2015 | Italian | Cohort | 16.5 | 35–69 | 391/10786 | 420.56 VS 198.55 | 0.65(0.44-0.95) | height, waist hip ratio, age at menarche, menopausal status, oral contraceptive use, parity, education, family history of breast cancer, energy intake and alcohol intake. |
| Bassett, J. K.2013 | Melbourne | Cohort | 16 | 27–80 | 936/20756 | 422 VS 224 | 0.99(0.83-1.19) | ethnicity, menopausal status, age at menarche, parity and lactation, oral contraceptive use, hormone replacement therapy use, physical activity, alcohol consumption, smoking status, education level and BMI. |
| Shrubsole, M. J.2011 | Shanghai | Cohort | 9.2 | 40-70 | 718/74,942 | 404 VS 194 | 0.79(0.59-1.06) | age at baseline, age at menarche, parity, age at first livebirth, educational attainment, physical activity, use of a B vitamin supplement, height, and total daily intakes of energy, vegetables, and fat, menopausal status. |
| Stevens, V. L.2010 | Americe | Cohort | 13.5 | 50-74 | 3898/70,656 | ≥918.9 VS <192.2 | 1.03(0.93-1.15) | age, includes alcohol use, multivitamin use, race, education, first-family history of breast cancer, history of breast lump, hormone replacement therapy, parity and age at first birth, age at menarche, age at menopause, physical activity, BMI, and energy. |
| Roswall, N.2010 | Danish | Cohort | 10.6 | 50–64 | 1072/26224 | >463.9 VS <288.2 | 1.23(0.97-1.56) | total intake of the three other micronutrients as well as dietary intake for the supplemental intake and supplemental intake for the dietary intake and further for alcohol intake, body mass index, hormone replacement therapy (HRT) use, duration of HRT use, number of births, parity/ nulliparity, age at first birth and school education. |
| Maruti, S. S.2009 | Washington | Cohort | 6 | 50-76 | 743/35023 | 1302 VS 325 | 0.90(0.72-1.12) | age, race, family history of breast cancer, mammography within2y preceding baseline, history of breast biopsy, age at menarche, age at first birth, age at menopause, years of combined estrogen and progestin postmenopausal hormone use, BMI, height, total physical activity, and alcohol intake in the past year. |
| Duffy CM.2009 | USA | Cohort | <10 | 50-79 | 1783/88530 | >642 VS <227.6 | 0.97(0.84-1.12) | tobacco consumption, BMI, history of breast biopsy, number of pregnancies, ever breast fed, family history, previous combined HRT use, age at menarche, age at menopause, weekly METs. |
| Larsson, S. C.2008 | Swedish | Cohort | 17.4 | ≥45 | 2952/61433 | ≥277 VS <200 | 1.01 (0.90-1.13) | age, education, body mass index, height, parity, age at first birth, age at menarche, age at menopause, use of oral contraceptives, use of postmenopausal hormones, family history of breast cancer, history of benign breast disease, and intakes of alcohol and total energy. |
| Kabat, G. C.2008 | Canada | Cohort | 16.4 | 40-59 | 2491/89835 | >374 VS <237 | 1.02 (0.90-1.17) | age, body mass index, pack-years of smoking, years of education, menopausal status, family history of breast cancer, history of breast biopsy, age at menarche, parity, oral contraceptive use, hormone replacement therapy, and intake of calories. In addition, all nutrients except alcohol were adjusted for alcohol intake (continuous). |
| Ericson, U.2007 | Malmo | Cohort | 9.5 | ≥50 | 392/11699 | 456 VS 160 | 0.56 (0.34-0.91) | age, method version, season, and total energy, weight, height, leisure-time physical activity, household work, smoking, socioeconomic status, age at menopause, hormone replacement therapy, and alcohol intake. |
| Cho, E.2007 | USA | Cohort | 12 | 26-46 | 1032/90663 | 822 VS 237 | 1.09 (0.88-1.34) | age and smoking, height, parity and age at first birth, body mass index, age at menarche, family history of breast cancer, history of benign breast disease, oral contraceptive use, and intakes of alcohol, energy, and animal fat. |
| Stolzenberg-Solomon RZ.2006 | USA | Cohort | 4.94 | 55-74 | 691/25400 | >853.0 VS ≤335.5 | 1.32(1.04-1.68) | energy, hormone replacement therapy, education, and BMI. |
| Lajous, M.2006 | French | Cohort | 9 | postmenopausal | 1812/62,739 | 522 VS 296 | 0.78(0.67-0.90) | age, two-year follow-up period, region of residence, years of education, family breast cancer, history of benign breast disease (yes or no), age at menarche, parity, breast feeding, years since last use of oral contraceptives, age at menopause, years of hormone replacement therapy use, regular mammographic evaluation, height in cm, body mass index, vitamin supplement use, alcohol intake and physical activity. |
| Feigelson, H. S.2003 | USA | Cohort | 5 | 40–87 | 1303/66,561 | >603.7 VS <209.8 | 1.10(0.94-1.29) | age, includes ethanol, dietary folate, methionine, multivitamin use, race, education, first-degree family history of breast cancer, history of breast lump, mammographic history, HRT use, parity and age at first birth, age at menopause, age at menarche, physical activity, BMI, adult weight gain, and energy. |
| Cho, E.2003 | USA | Cohort | 8 | 26-46 | 714/90655 | 826 VS 228 | 1.03(0.81-1.32) | age, smoking, height, parity and age at first birth, body mass index , age at menarche, family history of breast cancer, history of benign breast disease, oral contraceptive use, menopausal status, alcohol intake, energy, and animal fat. |
| Rohan TE.2000 | Canada | Cohort | >10 | 50-59 | 1469/56837 | 305.01-354.28 VS <224.78 | 0.99(0.79-1.25) | energy intake, age, age at menarche, number of live births, menopausal status, family history of breast cancer in a first-degree relative, practice of breast self-examination, alcohol consumption, randomization group, and study center. |
| Zhang, S. M.1999 | USA | Cohort | 16 | 30-55 | 3483/88818 | ≥600 VS <150 | 0.93(0.83-1.03) | age, length of follow-up, total energy intake, parity, age at first birth, age at menarche, history of breast cancer in mother or a sister, history of benign breast disease, alcohol intake, body mass index at age 18 years, weight change from age 18 years, height in cm, age at menopause, and postmenopausal hormone use. |
| Shana J. Kim1.2019 | Canada | Case–control | 1994-2016 | 18–70 | 129/271 | > 89.29 VS never | 0.54(0.27-1.10) | age, BRCA mutation type, BMI, alcohol consumption, regular smoker |
| Hatami, M.2019 | NA | Case–control | NA | NA | 151/154 | Q4 VS Q1 | 0.09(0.04-0.21) | Age, education, BMI. |
| Gong, Z.2014 | African American | Case–control | 2006-2012 | 20–75 | 749/833 | >636.4 VS ≤334.8 | 0.76(0.49-1.18) | age, ethnicity, country of origin, education, BMI, age at menarche, menopausal status, age at menopause, parity, age at first birth, breastfeeding status, family history of breast cancer, OC use, history of benign breast disease, HRT use, smoking status, alcohol consumption, and total energy intake. |
| Gong, Z.2.2014 | European American | Case–control | 2006-2012 | 20–75 | 744/690 | >636.4 VS ≤334.8 | 1.11(0.72-1.70) | age, ethnicity, country of origin, education, BMI, age at menarche, menopausal status, age at menopause, parity, age at first birth, breastfeeding status, family history of breast cancer, OC use, history of benign breast disease, HRT use, smoking status, alcohol consumption, and total energy intake. |
| Yang, D.2013 | USA | Case–control | 1999-2004 | 25-79 | 2325/2525 | >893 VS ≤405 | 0.90(0.64-1.26) | age, center, ethnicity, education, body mass index, total MET hours per week, total energy intake per day, total daily fiber intake, cigarette status, alcohol intake, parity, family history, oral contraceptive use and menopausal status. |
| Islam, T.2013 | Japan | Case–control | 2001-2005 | 20-79 | 1754/3508 | (359.4–1287.5) VS (130.9-278.5) | 0.79(0.68-0.93) | age, menopausal status, conditional logistic model for smoking habit, BMI, total nonalcohol energy, daily physical activity of any intensity, family history of breast cancer in first-degree relatives, menopausal status, age at menarche, parity, alcohol drinking and, referral pattern to our hospital. |
| Zhang, C. X.2011 | China | Case–control | 2007-2008 | 25-70 | 438/438 | >242.8 VS <155.7 | 0.32(0.21-0.49) | age at menarche, live births and age at first live birth, months of breast-feeding, BMI, history of benign breast disease, mother/sister/daughter with breast cancer, physical activity, passive smoking and total energy intake. |
| Lee, S. A.2010 | Korea | Case–control | 2001-2003 | 35-63 | 323/323 | >317.3 VS ≤190.3 | 0.5(0.3-0.9) | age, body mass index, education, age of first full-term pregnancy, family history of breast cancer, alcohol consumption, pack year of cigarette smoking, and total energy intake. |
| Ma, E.2009 | Japan | Case–control | 2001-2005 | 20-74 | 405/405 | >500.7 VS <386 | 1.01(0.62-1.64) | BMI, education, smoking status (never, ever), alcohol con- sumption, age at menarche, age at first live birth in parous women, menstruation status, breast feeding, number of live births, and moderate physical activity in the preceding 5 yr. |
| Lin, J.2008 | USA | Case–control | 1993-2004 | ≥45 | 848/848 | >582 VS ≤263.9 | 1.24(0.88-1.76) | matching variables, age, randomized treatment assignment, BMI, family history of breast cancer in a first-degree relative, history of benign breast disease, smoking, physical activity, alcohol consumption, age at menarche, age at menopause, parity, and age at first birth. |
| Tjønneland, A.2006 | Danish | Case–control | 1993-2000 | 50-64 | 388/388 | >400 VS ≤250 | 0.60(0.35-1.06) | vitamin C, total energy, school education, body mass index, parous/nulliparous and number of births, age at birth of first child, history of benign breast tumor surgery. |
| Lajous, M.2006 | Mexican | Case–control | 1990-1995 | 23-87 | 475/1391 | 454 VS 224 | 0.64(0.45-0.90) | age, socioeconomic status, family history of breast cancer, menopausal status, parity, availability of BMI, total caloric intake, dietary fiber carbohydrate intake, and polyunsaturated fat intake. |
| Chen, J.2005 | New York | Case–control | 1996-1997 | NA | 1481/1518 | >722 VS ≤208 | 0.95(0.74-1.22) | age, family history of breast cancer in first-degree relative, history of benign breast disease, educational attainment, body mass index at age 20, and kilocalories per day. |
| Adzersen, K. H.2003 | Germany | Case–control | 1998-2000 | 25-75 | 310/353 | >147 VS <77 | 0.47(0.25-0.8) | age, total energy without alcohol intake, age at menarche for categories, age at first birth by categories never, age at menopause, mother/sister with breast cancer, current smoking, history of benign breast disease and/or operation, BMI, consumption of alcohol, current hormone replacement therapy or HRT during the past year . |
| Sharp, L.2002 | England | Case–control | 1998-1999 | 50-69 | 62/66 | >303.7 VS <255 | 0.49(0.20-1.20) | total energy intake. |
| Shrubsole, M. J.2001 | Shanghai | Case–control | 1996-1998 | 25-64 | 1321/1382 | Q5 VS Q1 | 0.62(0.46-0.82) | total energy, age, education, family history of breast cancer, personal history of fibroadenoma, age at menarche, parity, age at first live birth, menopausal status, age at menopause, physical activity, and waist: hip ratio, total fruit and vegetable intake and total animal food intake. |
| Levi, F.2001 | Swiss | Case–control | 1993-1999 | 23-74 | 289/442 | 359000 VS 189000 | 0.45(0.42-0.88) | age, education, parity, menopausal status, body mass index, total energy intake, and alcohol drinking. |
| Ronco, A.1999 | Uruguay | Case–control | 1994-1997 | 20-89 | 400/405 | Q4 VS Q1 | 0.7(0.46-1.07) | age, residence, urban/rural status, family history of breast cancer in a 1st-degree relative, body mass index, age at menarche, parity, menopausal status, and total energy intake plus each nutrient. |
| Potischman, N.1999 | USA | Case–control | 1990-1992 | 20-44 | 568/1451 | ≥327 VS <173 | 0.89(0.7-1.2) | age at diagnosis, study site, ethnicity, education,age at first birth, alcohol intake, years of oral contraceptive use and smoking status. |
| Thorand, B.1998 | Europe | Case–control | 1991-1992 | 38-80 | 43/106 | 271 VS 170 | 1.14(0.73-1.79) | age, BMI, exogenous hormone use, age at menarche, null parity, smoking status, SES. |

Abbreviations: OR, odds ratio; CI, confidence interval; BMI, body mass index; BRCA, breast cancer; HRT, hormone replacement therapy; MET, metabolic equivalent of energy; OC, oral contraceptive
